# Network analysis for the identification of hub genes and related molecules as potential biomarkers associated with the differentiation of bone marrow-derived stem cells into hepatocytes

**DOI:** 10.18632/aging.204344

**Published:** 2022-10-20

**Authors:** Ying-Hao Han, Xin-Mei He, Seung-Jae Lee, Ying-Ying Mao, Xuan-Chen Liu, Hu-Nan Sun, Mei-Hua Jin, Taeho Kwon

**Affiliations:** 1 College of Life Science and Technology, Heilongjiang Bayi Agricultural University, Daqing 163319, Heilongjiang, P.R. China; 2 Functional Biomaterial Research Center, Korea Research Institute of Bioscience and Biotechnology, Jeongeup-Si 56212, Jeonbuk, Republic of Korea; 3 Department of Applied Biological Engineering, KRIBB School of Biotechnology, University of Science and Technology, Daejeon 34113, Republic of Korea; 4 Primate Resources Center, Korea Research Institute of Bioscience and Biotechnology (KRIBB), Jeongeup-Si 56216, Jeonbuk, Republic of Korea; 5 Department of Functional Genomics, KRIBB School of Bioscience, University of Science and Technology, Daejeon 34113, Republic of Korea

**Keywords:** hepatocytes, BMSCs, hub genes, miRNA, lncRNA

## Abstract

The incidence of liver diseases has been increasing steadily. However, it has some shortcomings, such as high cost and organ donor scarcity. The application of stem cell research has brought new ideas for the treatment of liver diseases. Therefore, it is particularly important to clarify the molecular and regulatory mechanisms of differentiation of bone marrow-derived stem cells (BMSCs) into liver cells. Herein, we screened differentially expressed genes between hepatocytes and untreated BMSCs to identify the genes responsible for the differentiation of BMSCs into hepatocytes. GSE30419 gene microarray data of BMSCs and GSE72088 gene microarray data of primary hepatocytes were obtained from the Gene Expression Omnibus database. Transcriptome Analysis Console software showed that 1896 genes were upregulated and 2506 were downregulated in hepatocytes as compared with BMSCs. Hub genes were analyzed using the STRING and Cytoscape v 3.8.2, revealing that twenty-four hub genes, play a pivotal role in the differentiation of BMSCs into hepatocytes. The expression of the hub genes in the BMSCs and hepatocytes was verified by reverse transcription-quantitative PCR (RT-qPCR). Next, the target miRNAs of hub genes were predicted, and then the lncRNAs regulating miRNAs was discovered, thus forming the lncRNA-miRNA-mRNA interaction chain. The results indicate that the lncRNA-miRNA-mRNA interaction chain may play an important role in the differentiation of BMSCs into hepatocytes, which provides a new therapeutic target for liver disease treatment.

## INTRODUCTION

A liver transplant is a significant way to treat patients with severe liver damage, such as decompensated cirrhosis, liver failure, and advanced liver cancer [1]. However, there is a scarcity of liver donors, and transplantation is associated with immune rejection and other problems [2]. Over the past few decades, in addition to advances in biological treatment research, molecular biology, cell bioengineering, and the stem cell research, stem cell therapy has emerged as an economic and feasible liver disease treatment for the end-stage liver disease [3], particularly decompensated cirrhosis liver failure and advanced liver cancer, and offers an effective strategy with no limit of supply and demand [3]. Stem cell therapy has broad application prospects in liver disease [4]. Bone marrow-derived stem cells (BMSCs) are widely used as adult stem cells, which originate from the mesoderm [5, 6]. Several experiments have demonstrated that BMSCs can differentiate into the cells of the mesoderm lineage, such as osteoblasts [7], adipocytes [8], muscle cells [9], neurons and brain cells [10], cardiomyocytes [11], and hepatocytes [12]. Growing evidence suggests that BMSCs can differentiate into hepatocytes, presenting interesting possibilities for cellular therapy of liver diseases. Previous reports have shown that decreased Wnt signaling contributes to the differentiation of BMSCs into hepatocytes [13]. Another study demonstrated the ability of BMSCs to differentiate into liver cells [14]. In addition, Kang et al. reported that rat BMSCs differentiate into hepatocytes [15]. It has been reported that cytokines, such as HGF and bFGF, are key contributing factors in promoting cell differentiation. Under certain conditions, HGF and bFGF can promote BMSCs differentiation into liver cells for the treatment of advanced liver disease [16, 17].

Owing to the emergence and development of RNA-sequencing technology, a large number of miRNA, lncRNA, and circRNA have been discovered and utilized [18]. LncRNA and miRNA are the two most important types of ncRNA. MiRNAs exhibit post-transcriptional inhibitory effects in animals and plants by pairing with target mRNAs [19]. LncRNA is a class of ncRNAs that does not encode proteins and whose transcripts are longer than 200 nt [20]. LncRNAs are speculated to regulate protein-coding genes in several ways. Studies have found that lncRNA regulates miRNA in three ways: (1) as a precursor or host of miRNAs; (2) lncRNAs and miRNAs compete for mRNA binding; and (3) LncRNA acts as a molecular sponge by absorbing miRNA, thereby regulating gene transcription and expression. Furthermore, miRNA and lncRNA play a major role in various life activities, and in the occurrence and development of liver disease. Most importantly, these are potential therapeutic targets and diagnostic biomarkers.

At present, the mechanism underlying the differentiation of BMSCs into hepatocytes is unclear. Further investigation of miRNA and lncRNA can advance the research of the differential genes. Therefore, we first analyzed the hub genes in the differentiation process of BMSCs into hepatocytes, and further identified miRNAs and lncRNAs, namely, miR-186, miR-703, miR-466k, miR-23a, miR-692, miR-466l, miR-137, miR-383, miR-466d-5p, miR-23b, miR-539, Zfp469, 1700020I14Rik, Gm42418, Zfas1, Dubr, and Peg13 as potential biomolecules.

The chip data for this study was obtained from a comprehensive gene expression database, and the differential genes were analyzed using bioinformatics software. Different genes were screened to obtain the hub genes that control the differentiation of BMSCs into hepatocytes and liver development. These hub genes include Cat, Cyp2e1, Pah, Ugt2a3, Acss2, Aldh6a1, Hmgcs2, H6pd, Aldh1a7, Hmgcl, Ugt1a1, Arg1, Otc, Baat, Slco1b2, Onecut1, Hhex, Proc, Cdk4, Il6, Fn1, Erbb2, Ccnd1, and Bmp4. In summary, this study provides potential therapeutic targets for the treatment of liver diseases.

## RESULTS

### DEG screening

**Figure 1:**
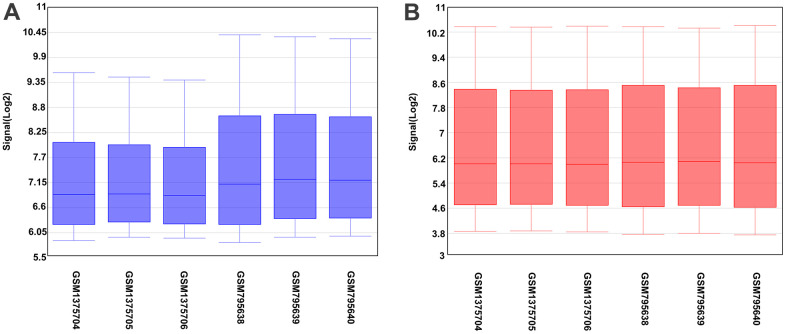
**Box plot of sample data.** The abscissa of the box plot indicates the sample names and the ordinate represents the sample expression value. (**A**) Before the standardization. (**B**) After the standardization.

Figure 1A shows differences in the data, indicating unstandardized data, while Figure 1B shows roughly well-standardized data. A total of 4402 differential genes were detected in hepatocytes, with 1896 upregulated and 2506 downregulated genes compared with BMSCs.

### Principal-component analysis (PCA)

**Figure 2:**
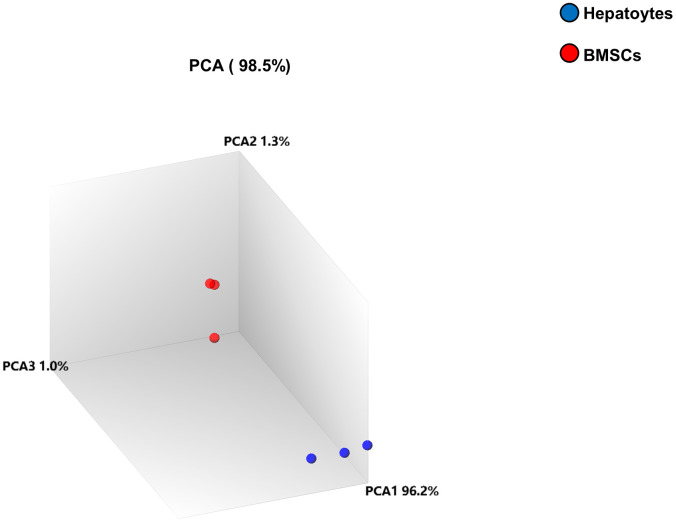
**Principal-component analysis (PCA).** PCA was performed between the two samples. The differently colored circles indicate the two different samples. The blue and red circles denote the hepatocyte samples and BMSC samples, respectively. The contribution of PCA1, PCA2, and PCA3 to the total mapped difference (98.5%) is 96.2%, 1.3% and 1.0%, respectively.

The principal-component analysis (PCA) was utilized to obtain information about the overall composition of the analyzed complex microarray datasets. The three samples in the hepatocyte sample group were closely distributed, indicating their high mutual similarity. The BMSCs group exhibited high repeatability. However, the dispersion between the hepatocyte sample and BMSCs groups was very high and well distinguishable, with distinct differences between them, as shown in Figure 2.

### Heat map and volcano plot analysis of DEGs

**Figure 3:**
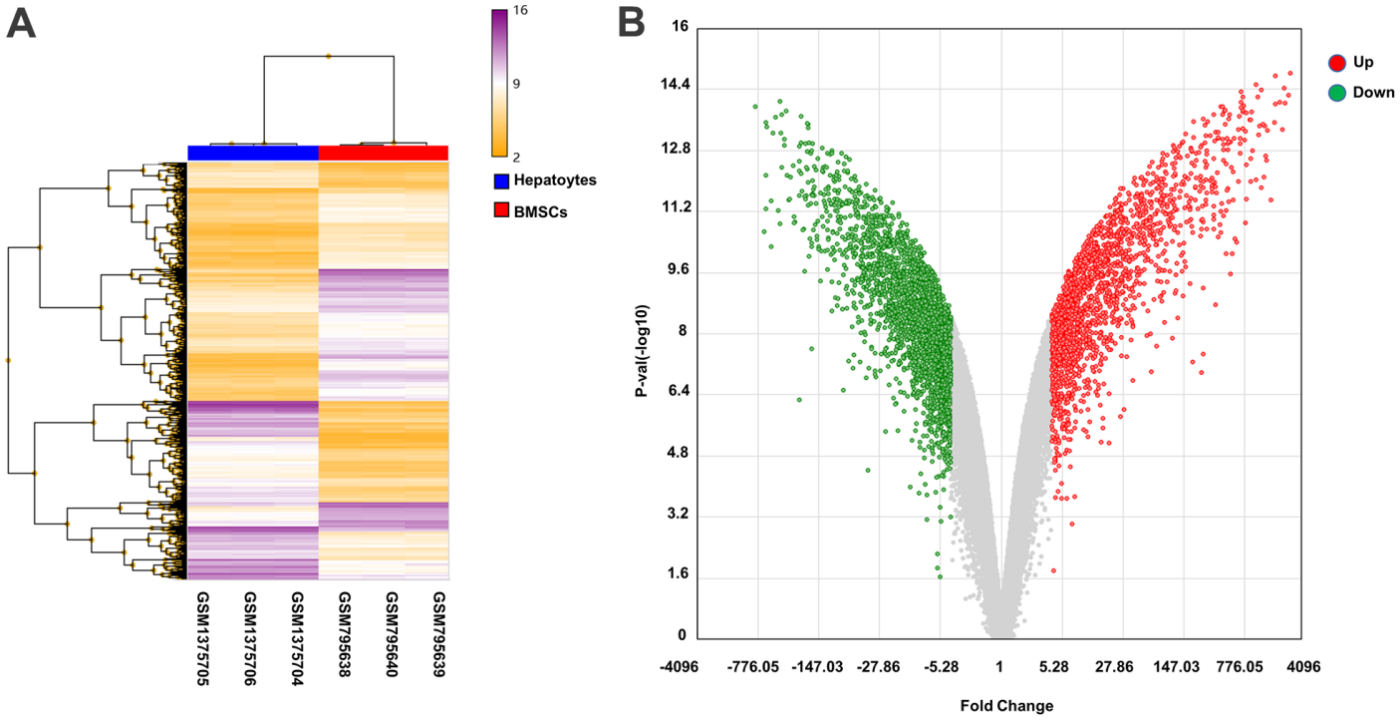
**Heat map and volcano plot analysis of DEGs.** (**A**) Heat map of DEGs. The abscissa shows the clustering of samples: GSM795638, GSM795639, and GSM795640 are BMSCs samples, and GSM1375704, GSM1375705, and GSM1375706 are hepatocyte samples. The right vertical axis indicates the clustering of genes in the two samples. The main body of the heat map contains genes, with purple and yellow lines denoting upregulated and downregulated genes, respectively. (**B**) Volcano plot of DEGs. The red and green dots represent upregulated and downregulated genes, respectively, in hepatocytes samples.

The abscissa represents the sample data, which can be divided into two categories: BMSCs and hepatocytes. In addition, the expression of genes in the BMSCs and hepatocytes is shown in Figure 3A.

The log2-fold change difference and the negative logarithm of *p* values between the volcano map samples of DEGs in the BMSC and hepatocyte samples are indicated on the X and Y axes, respectively, each point representing a single gene with detectable expression in both samples. The down-regulated and up-regulated genes were indicated by blue and red, respectively, and insignificant genes were indicated by gray dots. As compared to BMSCs, 1896 and 2506 genes were upregulated and downregulated, respectively, in hepatocytes (Figure 3B).

### GO analysis of DEGs

**Figure 4:**
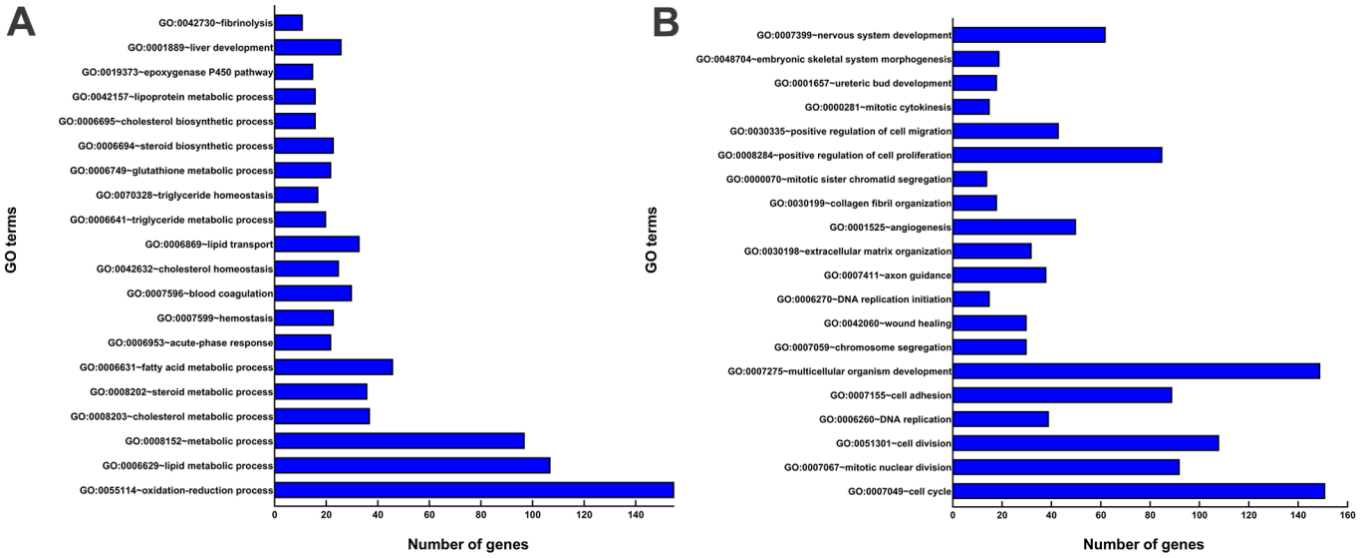
**GO enrichment analyses of DEGs.** (**A**) The top 20 GO terms enriched for the upregulated genes. (**B**) The top 20 GO terms enriched for the downregulated genes. The x-axis shows the number of genes contained in GO terms. The y-axis shows GO terms with significantly enriched DEGs.

All DEGs were analyzed by DAVID software (https://david.ncifcrf.gov/tools.jsp). GO enrichment analysis showed that the following GO terms were included for upregulated genes: oxidation-reduction process (GO:0055114), lipid metabolic process (GO:0006629), metabolic process (GO:0008152), and liver development(GO:0001889) (Figure 4A), and the following for downregulated genes: regulation of cell cycle (GO:0007049), cell division (GO:0051301), multicellular organism development (GO:0007275), and positive regulation of cell proliferation (GO:0008284) (Figure 4B).

### Construction of PPI networks of oxidation-reduction process (GO:0055114), metabolic process (GO:0008152), liver development (GO:0001889), and positive regulation of cell proliferation (GO:0008284) biological process

**Figure 5:**
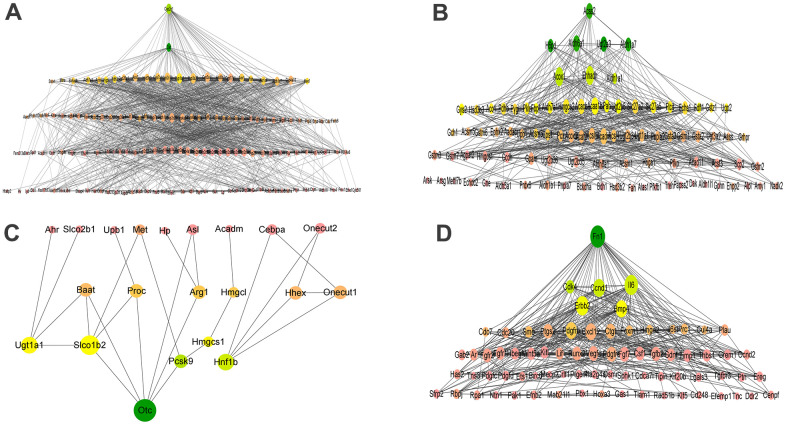
PPI networks of oxidation-reduction process (GO:0055114, **A**), metabolic process (GO:0008152, **B**), liver development (GO:0001889, **C**), and positive regulation of cell proliferation (GO:0042127, **D**) biological process. (**A**) PPI networks of oxidation-reduction process. (**B**) PPI networks of metabolic process. (**C**) PPI networks of liver development. (**D**) PPI networks of positive regulation of cell proliferation. The node size was determined according to the degree of connectivity. The color gradient of nodes reflects the change in BC, wherein the change of green to yellow coincides with its change from high to low.

To screen out the hub genes during the differentiation of BMSCs into hepatocytes, up-regulated genes in hepatocyte oxidation-reduction process, hepatic metabolism process and liver development, and down-regulated genes in positive regulation of cell proliferation were selected because they are closely related to hepatocyte differentiation. The PPI network was constructed with four GO genes (Table 1) and STRING database (https://www.string-db.org/). The DEGs contained in the four GO terms were respectively uploaded to STRING to construct a PPI network. The data exported from the STRING was screened for the hub genes. Two genes were valuable in the oxidation-reduction process of hepatocytes: Cat and Cyp2e1. Seven genes were shown to be hub genes within the metabolic process: Pah, Ugt2a3, Acss2, Aldh6a1, Hmgcs2, H6pd, and Aldh1a7. Nine genes play a critical role in liver development: namely Hmgcl, Ugt1a1, Arg1, Otc, Baat, Slco1b2, Onecut1, Hhex, and Proc; and six genes in suppressing liver differentiation: Cdk4, Il6, Fn1, Erbb2, Ccnd1 and Bmp4 (Table 2). Cytoscape v.3.8.2 software act as a pathway for PPI network mapping and analysis. Degree and betweenness centrality (BC) value represents the numbers of interactions of a particular protein and represents nodes passing the node in the shortest distance, respectively. In the final network shown in Figure 5, genes with larger nodes and darker colors are more essential in the PPI network.

**Table 1:** Genes in GO terms of liver development and regulation of cell proliferation.

**GO terms**	**Biological process**	**Genes**
GO:0055114	Oxidation-reduction process	DMGDH/ALDH1L1/STEAP4/GLDC/CYP3A11/CYP3A13/H2-KE6/MSMO1/CMAH/TM7SF2/CYP2D9/CYP2D10/TDO2/FADS6/IYD/PHYHD1/CYP2C70/ACAD11/PHYH/HGD/DIO1/DHDH/PIPOX/KMO/CYP2D26/CYP27A1/CYP39A1/POR/ETHE1/AKR1C14/FDX1/GPD1/AKR1C13/CYP2E1/HPD/ALDH7A1/GCDH/CYP4A12A/H6PD/MAOB/MGST1/SDR39U1/HSD11B1/ADH4/ADH1/AGMO/LDHD/PNPO/HAO1/DECR2/PRODH/FDFT1/SDR42E1/CYP2J5/IDH1/ADHFE1/DHCR24/CYP3A25/CYP2C29/CP/PRDX6/BC026585/CYP4F14/QDPR/CYP26A1/GLUD1/MSRA/GRHPR/ALDH6A1/PAH/CYP1A2/PIR/NOX4/DHCR7/KDM5D/SRXN1/RDH7/CYP2C39/CYP2C38/AKR1B7/CYP2C37/CYP2B10/AOX3/CYP4V3/CYP2R1/ME1/AOX1/SCD1/ACADM/AASS/HTATIP2/BCKDHA/CBR1/HSDL2/CYP4A10/CYP3A44/SORD/CYP4A14/CYP7B1/ALDH5A1/ACOX2/BDH1/ACOX1/EHHADH/CYP3A41A/CHDH/DPYD/CAT/ALDH1A1/BLVRB/ALDH1A7/HPGD/HAAO/AKR1D1/HSD17B13/HSD17B6/PDHB/HMGCR/HSD17B7/CYP7A1/DDO/CYP17A1/CRYZ/GULO/ALDH1B1/CYP2C55/CYP2A12/CYP2C54/CREG1/HSD17B2/ASPDH/SC5D/CYP2C50/BCO2/CYP2F2/BCO1/CRYM/AAED1/HSD3B7/EGLN3/HSD3B2/SMOX/HSD3B3/GSR/CYP4B1/FMO1/BBOX1/FMO4/FAM213A/FMO5/AKR1C6/ALDH8A1/CDO1/CYB561/UOX
GO:0008152	Metabolic process	ALDH1L1/ ACSM3/ ACSM1/ ACSM5/ SCP2/ ENPP2/ ACAD11/ ACSL1/ ACSL4/ HOGA1/ UPB1/ AMY1/ SLC27A2/ ALDH7A1/ SLC27A5/ GNE/ PFKFB1/ GCDH/ H6PD/ ACACB/ ACAT2/ TKFC/ ARSK/ UGT3A2/ ARSG/ FDFT1/ GSTM3/ GSTM2/ GSTM1/ EPHX2/ ACSF3/ PRDX6/ PM20D1/ GRHPR/ ALDH6A1/ CPS1/ PAH/ MYO5B/ ALPL/ ECHDC2/ ECHDC3/ ACO1/ GSTM7/ GSTM6/ ALAS1/ TREH/ PYGL/ IDE/ GPHN/ FTCD/ GYS2/ NADK2/ HYAL1/ UGT2A3/ HMGCS2/ ACADM/ AASS/ BCKDHA/ HMGCS1/ UGT1A1/ MAN1A/ ALDH5A1/ ACOX2/ BDH1/ ACOX1/ EHHADH/ ALDH1A1/ UGT2B5/ FBP1/ ALDH1A7/ ECHS1/ ACSS2/ PCX/ ACAA1B/ PDHB/ AGPAT2/ PAPSS2/ UGP2/ ALDH1B1/ METTL7B/ UGT2B34/ PKLR/ PNPLA7/ HSD3B2/ GCH1/ UGT2B36/ HSD3B3/ UGT2B35/ FAH/ GSTZ1/ GPAM/ AADAC/ GSTA3/ GSTA2/ ALDH8A1/ PFKP/ PNPLA2
GO:0001889	Liver development	CEBPA/ONECUT2/ONECUT1/HP/PCSK9/AK4/AHR/HMGCL/HHEX/SLCO1B2/SLCO2B1/GNPNAT1/ASL/ACADM/UGT1A1/CADM1/HMGCS1/ARG1/HNF1B/LSR/BAAT/UPB1/QDPR/PROC/MET/OTC
GO:0008284	Positive regulation of cell proliferation	CSF1/CDCA7L/RTKN2/TNC/FOXM1/RBPJ/ETS1/CTGF/CDC20/MECP2/LGALS3/FGF7/CCND2/EFEMP1/CCND1/PLAU/HOXA3/TIMP1/TNS3/PDGFRB/PDGFRA/TIPIN/TSC22D1/SPHK1/WNT5A/PLA2G4A/EMP2/SOX11/GAB2/OSMR/RUNX2/EREG/RAD51B/TGFBR3/GREM1/AR/TIAM1/SFRP2/SMO/GAS1/BIRC6/KIF20B/IL6ST/DDR2/HBEGF/PTGER4/NLGN2/EIF5A2/PTN/PTGS2/NTN1/THBS1/EFNB2/PAK1/PDGFD/ERBB2/PDGFC/HAS2/ARNT2/TGFB2/MARCKSL1/RPA1/FN1/LIF/HMGA2/VEGFC/CDC7/PBX1/CUL4A/BMP4/IL6/CENPF/KLF5/CXCL12/GDNF/CDK4/KITL/PRC1/MAB21L1/BCL2/CD248/SSBP3/CRLF1/FGFR2/FGFR1

**Table 2:** Hub genes selected based on visualize parameters like BC and degree.

**Network name**	**Gene**	**Degree**	**BC**	**AD**
Oxidation-reduction process	Cat	40	0.119805098	17.90
	Cyp2e1	50	0.062124918	
Metabolic process	Pah	17	0.070184791	10.37
	Ugt2a3	15	0.065342994	
	Acss2	20	0.060943054	
	Aldh6a1	18	0.059708481	
	Hmgcs2	20	0.053147572	
	H6pd	21	0.119700331	
	Aldh1a7	11	0.069738078	
Liver development	Hmgcl	3	0.257142857	2.57
	Ugt1a1	4	0.257142857	
	Arg1	3	0.133333333	
	Otc	5	0.393650794	
	Baat	4	0.400000000	
	Slco1b2	5	0.320634921	
	Onecut1	3	0.500000000	
	Hhex	3	0.500000000	
	Proc	4	0.215873016	
Positive regulation of cell proliferation	Cdk4	14	0.107299978	10.59
	Il6	34	0.145058759	
	Fn1	38	0.176673936	
	Erbb2	27	0.076760508	
	Ccnd1	31	0.180939463	
	Bmp4	28	0.137307941	

BC, Betweenness centrality; AD, average degree of all nodes.

### Hub genes detected in BMSCs and hepatocytes by RT-qPCR

**Figure 6:**
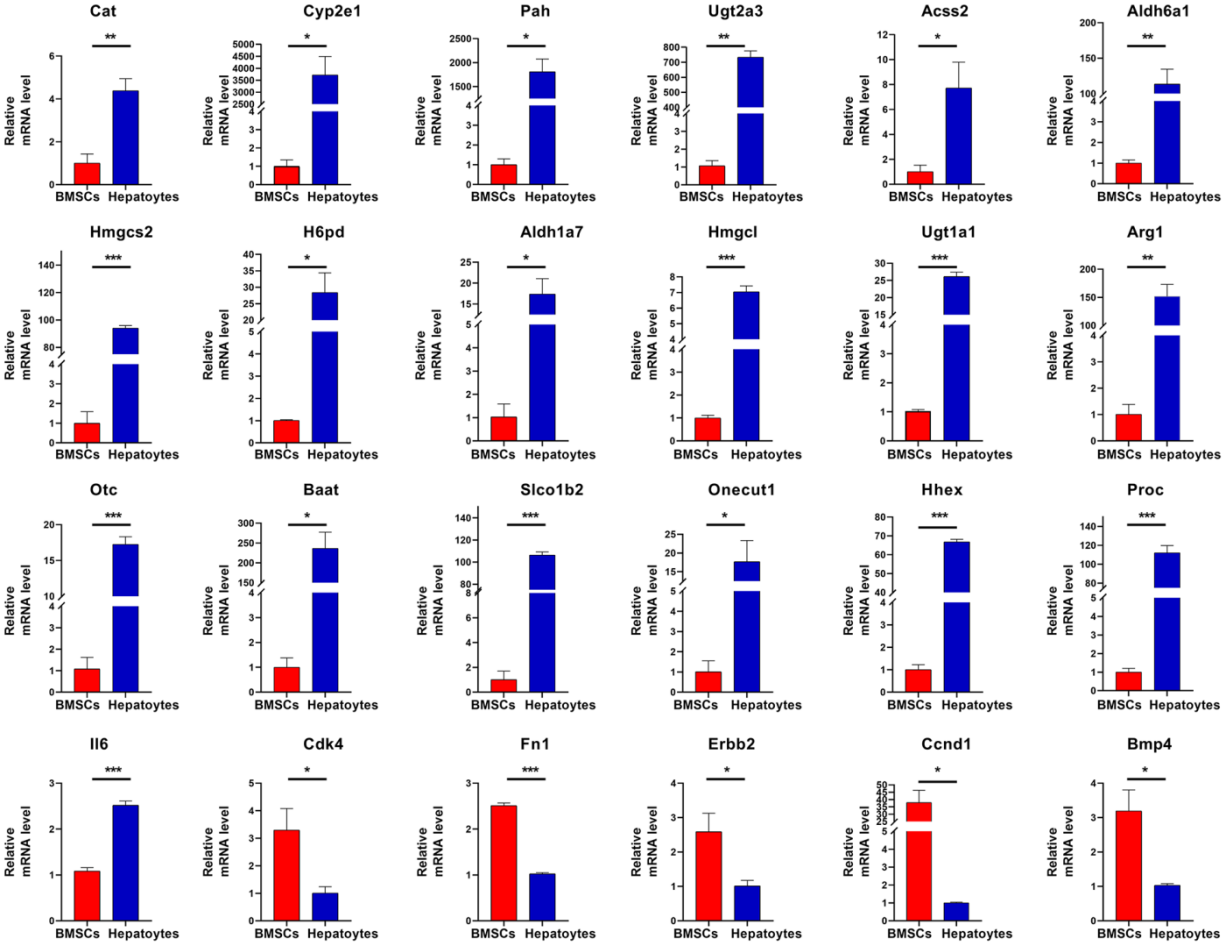
**RT-qPCR validation.** The expression of twenty-four hub genes was assessed. The hub genes are Cat, Cyp2e1, Pah, Ugt2a3, Acss2, Aldh6a1, Hmgcs2, H6pd, Aldh1a7, Hmgcl, Ugt1a1, Arg1, Otc, Baat, Slco1b2, Onecut1, Hhex, Proc, Il6,Cdk4, Fn1, Erbb2, Ccnd1, and Bmp4. Significance is shown by *P<0.05, **P<0.01 and ***P<0.001.

To investigate key molecular targets regulating differentiation of BMSCs into hepatocytes, four GO terms (Table 1) related to liver development and differentiation were selected. Twenty-four DEGs were identified as hub genes in the differentiation of BMSCs into hepatocytes. Then, twenty-four hub genes were verified by RT-qPCR. Twenty-three hub genes, namely Cat, Cyp2e1, Pah, Ugt2a3, Acss2, Aldh6a1, Hmgcs2, H6pd, Aldh1a7, Hmgcl, Ugt1a1, Arg1, Otc, Baat, Slco1b2, Onecut1, Hhex, Proc, Cdk4, Fn1, Erbb2, Ccnd1 and Bmp4 were consistent with RNA sequencing results, while IL6 gene was contrary to RNA sequencing results. All the 24 hub genes were differentially expressed. Together, these results suggested that twenty-four hub genes are important to regulate differentiation of BMSCs into hepatocytes (Figure 6).

### miRNA prediction

**Figure 7:**
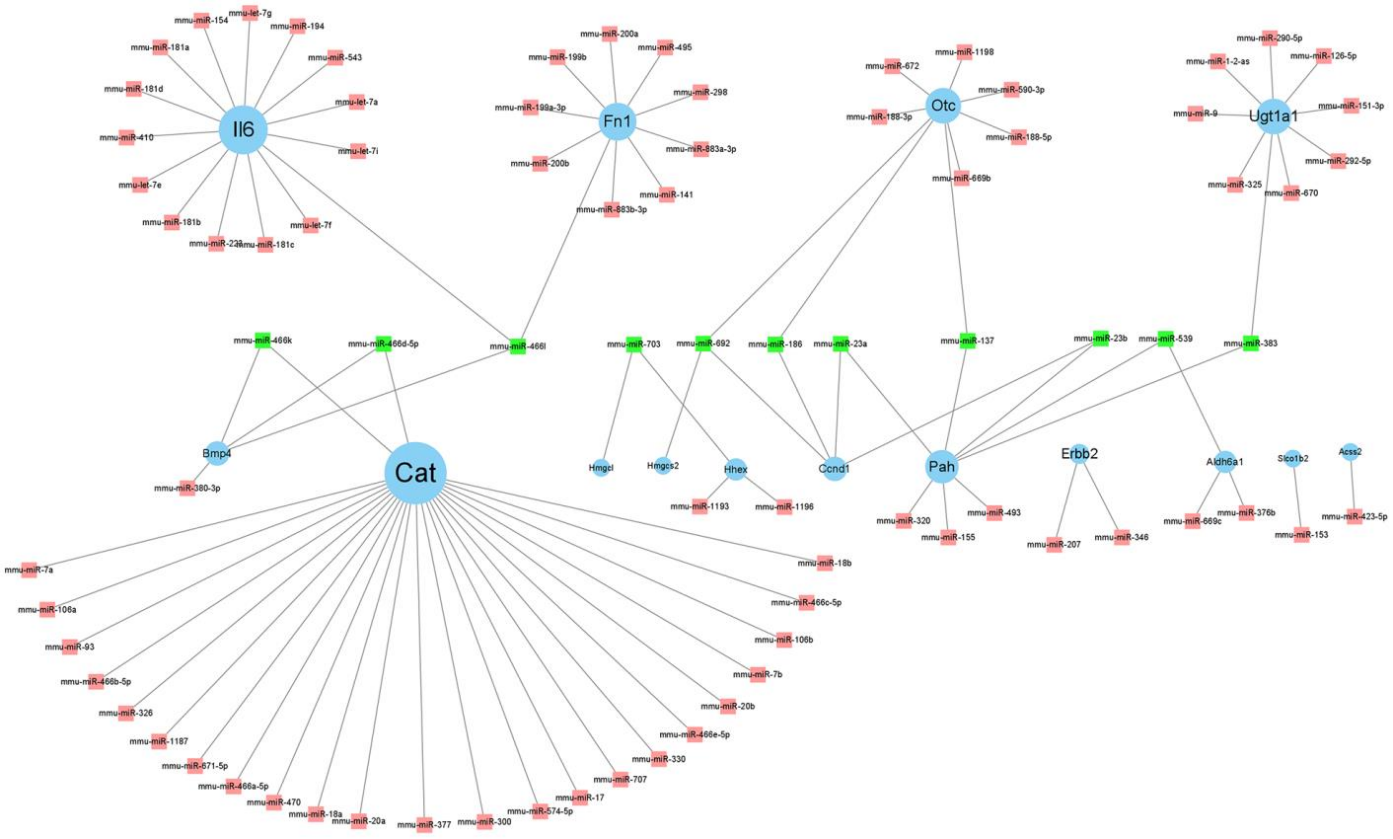
**Regulatory network of hub genes and its related miRNAs.** Cytoscape v. 3.8.2 software was used to visualize the relationship between genes and their targeted miRNAs. The blue circle nodes denote the genes, and the pink square nodes denote the miRNAs. MiRNAs targeting at least two genes are shown in green.

We predicted the miRNAs corresponding to the twenty-four hub genes in miRWalk (http://zmf.umm.uni-heidelberg.de/apps/zmf/mirwalk/predictedmirnagene.html). The screening criteria included the miRNA binding site should be in the 3'-UTR, the minimum nucleotide length of miRNA should be 7-mer, and P <0.05. Five prediction programs, miRanda, miRDB, miRWalk, RNA22, and TargetScan, were selected, and the intersection of their predictions was the final prediction result [21]. The genes and MiRNAs interaction network are shown in Figure 7. MiRNAs targeting at least two genes were counted (Table 3).

**Table 3:** miRNAs and its target genes.

**miRNA**	**Genes targeted by miRNA**	**Gene count**
miR-692	Otc,Hmgcs2,Ccnd1	3
miR-466l	Bmp4,Fn1,Il6	3
miR-703	Hhex,Hmgcl	2
miR-137	Otc,Pah	2
miR-186	Otc,Ccnd1	2
miR-383	Ugt1a1,Pah	2
miR-539	Aldh6a1,ah	2
miR-23a	Pah,Ccnd1	2
miR-23b	Pah,Ccnd1	2
miR-466d-5p	Cat,Bmp4	2
miR-466k	Cat,Bmp4	2

### lncRNA prediction

**Figure 8:**
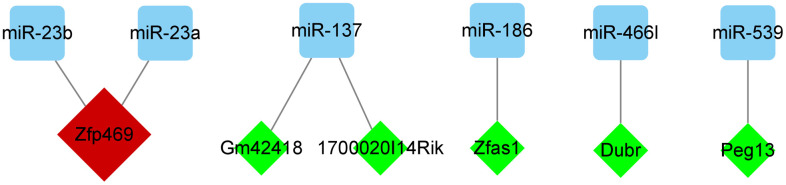
**Regulatory network of miRNAs and its targeted lncRNAs.** Cytoscape v. 3.8.2 software was used to visualize the relationship between miRNAs and their targeted lncRNAs. The green diamond nodes denote the lncRNAs and blue square nodes denote the miRNAs. LncRNAs targeting two miRNAs are shown in red.

In StarBase (https://starbase.sysu.edu.cn/), we predicted the lncRNAs corresponding to miR-692, miR-466l, miR-703, miR-137, miR-186, miR-383, miR-539, miR-23a, miR-466d-5p, miR-23b, and miR-466k. Very high stringency (≥3) was used as the prediction criterion. Only six miRNAs, namely miR-23a-3p, miR-23b-3p, miR-137-3p, miR-186-5p, miR-466l-3p, and miR-539-5p, met the criteria. Moreover, Zfp469 was observed to target two key miRNAs (Figure 8 and Table 4).

**Table 4:** lncRNAs and its target miRNAs.

**lncRNA**	**MiRNAs targeted by lncRNA**	**MiRNA count**
Zfp469	miR-23a,miR-23b	2
1700020I14Rik	miR-137	1
Gm42418	miR-137	1
Zfas1	miR-186	1
Dubr	miR-466l	1
Peg13	miR-539	1

## DISCUSSION

Liver disease is a serious hazard to human health. Ideal clinical intervention drugs and methods have not been identified yet in any part of the world. The liver transplantation is a conventional and effective intervention method. However, transplantation cannot meet the clinical needs due to the shortage of high-quality liver cells, allograft rejection, and other problems [22]. Therefore, there is an urgent need to develop new methods for liver disease intervention. Currently, stem cell-based cell replacement therapy has attracted worldwide attention. BMSC transplantation provides a new way to intervene liver disease. Several studies have shown that transplanted BMSCs can differentiate into hepatocytes to replace the function of damaged hepatocytes and tissues in liver due to their directional differentiation ability, promoting the recovery of liver injury. However, the mechanism of differentiation of BMSCs into hepatocytes remains unclear. The study of key genes and downstream regulatory mechanisms associated with the differentiation of BMSCs into liver cells has a profound clinical impact, which can provide potential targets for BMSC-based treatment of liver failure.

We uploaded chip data of BMSCs and hepatocytes to Transcriptome Analysis Console software for DEGs Analysis. Total 4402 DEGs were detected in hepatocytes, with 1896 upregulated and 2506 downregulated genes compared with BMSCs. GO analysis was performed on all the DEGs, and the results were as follows: among the upregulated genes, 11.44% were related to oxidation-reduction process, 7.16% to liver metabolism process, and 1.92% were to liver development process. Among the downregulated genes, 5.04% were related to cell proliferation. Oxidation-reduction process, metabolism process, liver development process, and cell proliferation are closely related to differentiation of hepatocytes.

The STRING search tool was used to build PPI network, and the hub genes were identified by analyzing the network. Among the hub genes, Cat and Cyp2e1 are related to the oxidation-reduction process of hepatocytes; Cat and FOXO3 are positively correlated with the differentiation of BMSCs into cells of the osteogenic lineage [23]. In the later stage of stem cells differentiation into hepatocytes, Cyp2e1 is expressed [24]. The hub genes Pah, Ugt2a3, Acss2, Aldh6a1, Hmgcs2, H6pd, and Aldh1a7 are related to liver metabolism. The mRNA level of Hmgcs2 increase during the differentiation process of embryonic stem cells to hepatocellular-like cells [25]. The role of Pah, Ugt2a3, Acss2, Aldh6a1, H6pd, or Aldh1a7 in hepatocyte cells is currently unclear. The hub genes Hmgcl, Ugt1a1, Arg1, Otc, Baat, Slco1b2, Onecut1, Hhex, and Proc are related to liver development. Ugt1a1 is a marker of hepatocytes, and its significant expression during the differentiation of human hematopoietic stem cells into hepatocytes indicates that HSCs have successfully differentiated into normal hepatocytes [26]. Arg1 expression is observed during hepatic-like phenotype differentiation of somatic stem cells *in vitro* [27]. In a previous study, increased Otc expression was reported in 201B7 cells after culture in hepatocyte differentiation initiation medium [28]. Onecut interacts with Lmx1a to promote the differentiation of ventral midbrain neural stem cells into dopamine neurons through the Wnt1-Lmx1a pathway [29]. Hhex regulates the hepatic differentiation of embryonic stem cells [30]. The role of Hmgcl, Baat, Slco1b2, or Proc in hepatocyte differentiation and development is unclear. The hub genes Cdk4, Il6, Fn1, Erbb2, Ccnd1, and Bmp4 regulate cell proliferation networks and are downregulated in hepatocytes compared with untreated BMSCs, suggesting inhibitory effects on liver differentiation. Studies have shown that expression of Cyclin B1 and Cdk4 during the hepatic differentiation of liver epithelial progenitor cells (LEPCs) induced by sodium butyrate may be related to the growth arrest of LEPCs shortly after treatment [31]. MSCs contribute to liver differentiation by activating the IL-6 / gp130-mediated STAT3 signaling pathway [32]. Ccnd1 has been reported to be associated with liver regeneration, and it is speculated that they play a key role in mouse hepatocytes [33]. Ccnd1 silencing suppresses liver cancer stem cells (LCSCs) differentiation [34]. Bmp4 is an important regulator of cell proliferation and differentiation. Studies have shown that Bmp4 is a key cytokine for the development of mouse embryonic stem cells into hepatocytes [35]. Fn1 and Erbb2 have not been reported in this respect. Furthermore, we verified twenty-four hub genes in the BMSCs and hepatocytes using RT-qPCR. All the twenty-four hub genes were differentially expressed.

We used miRWalk to predict upstream target miRNAs that regulate gene expression. Eleven miRNAs were predicted to target at least two hub genes each. Previous studies have reported that lncRNAs, located upstream of miRNAs, are not negligible in BMSCs differentiation; therefore, we used StarBase 2.0 for prediction. However, only miRNA-23a, miRNA-23b, miR-137, miRNA-186, miRNA-466l, and miRNA-539 were predicted to obtain target lncRNAs. Six lncRNAs, Zfp469, 1700020I14Rik, Gm42418, Zfas1, Dubr, and Peg13 were predicted by StarBase v2.0. Therefore, we introduced the above six miRNAs and their predicted lncRNAs in the regulation of BMSC differentiation. Studies have shown that down-regulation of miR-23a can promote osteogenic differentiation of BMSCs [36]. Mir-23b-3p can affect the hepatic trans-differentiation of MSCs [37]. Another study showed that miR-23b-3p can impact the differentiation of BMSCs [38], as do Mir-137-3p [39]. Current research shows that the relationship between miRNA-186-5p, miRNA-466l-3p, miRNA-539-5p, and BMSCs differentiation is not very clear. Among the 6 lncRNAs, only Zfas1 was found to be associated with BMSC differentiation, while the relationship between Zfp469, 1700020I14Rik, Gm42418, Dubr, Peg13, and BMSC differentiation remains unclear. Zfas1 has been reported to affect BMSC differentiation by sponging miR-499. However, further research is needed to confirm these results [40].

Taken together, through bioinformatics analysis, we identified key genes that regulate the differentiation of BMSCs into hepatocytes and their upstream miRNAs and lncRNAs, providing potential targets for stem-cell replacement therapy for liver diseases. Hence, exploring the key genes and downstream regulation mechanism of the differentiation of BMSCs into hepatocytes is essential for treating hepatic diseases.

## MATERIALS AND METHODS

### Acquisition of primary mouse BMSCs and hepatocytes

In this experiment, two-step perfusion method was used to isolate and acquire mouse primary hepatocytes. First, the liver was flushed with Ca2^+^-free HEPES, followed by HEPES perfusion containing collagenase and Ca2^+^, and then the cell suspension was cultured in DMEM/Ham’s F12 medium. The primary hepatocytes were established in complete DMEM/Ham’s F12 media placed in a 95% air/5% CO_2_, 37° C incubator [41].

The soft tissue on the surface of the mouse femur was carefully peeled off and the femur tissue was rinsed several times with a 1 mL syringe containing α-MEM culture solution, until it was transparent. Four rinsed femur cells were cultured in a medium containing BMSCs growth medium placed in a 95% air/5% CO_2_, 37° C incubator, and refreshed every three days [42–44].

### Download of microarray data

Gene Expression Omnibus (GEO) database provides gene expression profiles [45]. We downloaded two gene expression profiles of GSE30419 and GSE72088 from the GEO database datasets. GSE30419 included three untreated *Mus musculus* BMSC samples (GSM795638, GSM795639, and GSM795640), and dataset GSE72088 included three PBS treated *Mus musculus* primary hepatocytes samples (GSM1375704, GSM1375705, and GSM1375706).

### Screening of DEGs

The genes expression profiles of GSE30419 and GSE72088 were analyzed by Transcriptome Analysis Console software. | Fold Change | >4, P <0.05 and FDR <0.05 were used as the standard for analysis and screening of DEGs.

### GO enrichment analysis

To investigate the functions of these gene signatures, we performed GO enrichment analysis. The DAVID website has multiple functional enrichment for DEGs [46], and the screening criteria for GO functional enrichment is usually set as P <0.05 and FDR <0.05 [47]. The results were presented as bar graphs by Graph Pad Prism 6.0.

### Discovery of hub genes

The PPI network of differential genes was constructed using the STRING online platform and analyzed and processed by Cytoscape V.3.8.2 software [48]. The means of the degrees were calculated to screen out genes that showed a greater degree than the mean value. The hub genes with BC value greater than 0.05 and degree exceeds the mean were selected.

### Verification of hub genes expression using RT-qPCR

Total RNA was extracted from hepatocytes and BMSCs using TRIzol reagent (Sigma, St. Louis, MO, USA). We used the cDNA Synthesis Mix Kit (Innovagene, Hunan, China) to generate the first strand cDNA. Real-time PCR was implemented using SYBR Green qPCR Mix (Innovagene). Using the 2^−ΔΔCq^ method, we calculated and analyzed the expression situations of hub genes.

### Gene-miRNA prediction

We used the miRWalk website to predict target miRNAs located upstream to the genes [49]. Five databases including miRanda, miRDB, miRWalk, RNA22, and TargetScan were selected for prediction. The interaction between genes and miRNAs was visualized by Cytoscape V 3.8.2. MiRNAs targeting at least two genes were screened.

### miRNA-lncRNA prediction

Interaction between miRNAs and lncRNAs was identified using StarBase website [50], and Cytoscape V 3.8.2 was used for analysis and mapping. LncRNAs targeting multiple miRNAs are required to be carefully analyzed.

### Statistical analysis

Statistical analysis of the data was performed in IBM SPSS Statistics Version 26. The data based on RT-qPCR experiment was expressed as mean ± SD and presented using Graph Pad Prism 6.0. All the genes were tested in three separate experiments. Analysis was done using student’s unpaired t-tests. P<0.05 was statistically significant.
